# Impact of the Internet on Primary Care Staff in Glasgow

**DOI:** 10.2196/jmir.1.2.e7

**Published:** 1999-11-19

**Authors:** Steven M Wilson

**Keywords:** Internet, Consultation, General Practice, Information Technology, Patient Education

## Abstract

**Background:**

The Government has invested £7 million (approx. $11.5 million) to connect all Primary Care Practices in Scotland to the National Health Service Intranet (NHSnet). This provides General Practitioners (GPs) and Practice Nurses with access to the Internet and a wealth of healthcare information of varying quality.

**Objective:**

This study examines Primary Care Staff's use of the Internet, their views on the reliability of healthcare information available via the Internet, and their interaction with patients who have presented them with information downloaded from the Internet.

**Methods:**

A postal questionnaire was distributed to a random sample of 300 GPs and 130 Practice Nurses throughout Glasgow. There was a response rate of 60%.

**Results:**

Time restraints (20%) and concerns that they lack the necessary skills (17%) were highlighted as the most common reasons for not accessing the Internet. Sixty-nine per cent of GPs and 70% of Practice Nurses had looked at the Internet for healthcare information. Forty-eight per cent of GPs and 41% of Practice Nurses were concerned about the reliability of Internet information. Fifty-eight per cent of GPs and 34% of Practice Nurses have been approached by patients with Internet healthcare information. Sixty-five per cent of the information presented by patients was new to GPs.

**Conclusions:**

The majority of Primary Care Staff now have access to the Internet and use it to look up healthcare information. Almost half of GPs would consider referring their patients to the Internet for further information about their condition. Results highlight that the healthcare information downloaded from the Internet by patients is accurate, but patients have problems correctly interpreting this information. An increase in the use of home computers and free access to the Internet will see a continued increase in patients approaching GPs and Practice Nurses with healthcare information downloaded from the Internet.

## Introduction

The NHSnet was developed as part of a nationwide Information Management & Technology Strategy to create a National Health Service Intranet (NHSnet). The Government has now spent £7 million (approx. $11.5 million) to link every Primary Care Practice in Scotland to a secure connection to this network. This has important benefits for primary care practitioners. The Scottish Health Minister recently stated, "The GP (General Practitioner) will have at his fingertips a wealth of up-to-date information, new procedures, and the best of current thinking in the NHS" [1]. The use of this new technology and the knowledge of how to put it into practice are varied amongst Primary Care Staff [2,3].

A large number of good quality, credible healthcare resources are accessible using the Internet, allowing both patients and professionals to browse, download, and read endless reams of clinical information. This healthcare information exists in the form of online medical journals, Royal Colleges, national charities, pharmaceutical companies, disease support groups, etc. In addition to these high quality websites, there are many more less reputable websites, newsgroups, listservs, chat rooms, etc., containing medical information that has little or no scientific evidence. Although the Internet does not have a monopoly on misinformation, health information can be posted by anyone with access to the net and an interest in doing so. Impicciatore et al examined 40 web sites providing advice on the management of a feverish child and found that only four adhered to published guidelines [4].

The issues of misleading or inaccurate information are especially important in health care, as a little knowledge can be dangerous and very distressing for the less adept patient faced with the bare facts about his disease [6]. Therefore, it is important to know how patients use the Internet healthcare information available to them and the response of the GPs and Practice Nurses in Glasgow to caring for people with such information. Increasingly more patients are attending Primary Care Practices with health care information from the Internet in hand; this article looks at the impact on Primary Care Staff within Greater Glasgow.

## Methods

An anonymous postal questionnaire was sent to a randomly selected sample of 300 General Practitioners and 130 General Practice Nurses throughout Greater Glasgow. The names and addresses of Primary Care Staff were identified using data from Greater Glasgow Health Board Department of Public Health. A covering letter was included with the questionnaire outlining the aims of the study. Staff were questioned on their use of the Internet and its potential as a source of healthcare information. Respondents were asked if they were concerned about the reliability of Internet healthcare information, and on a scale of positive to uncomfortable were asked to rate their opinion on patients obtaining information from the Internet. A section was included asking Primary Care Staff about their interactions with patients holding Internet healthcare information. Finally, staff were asked if they would consider referring a patient to the Internet for further information, and if they would recommend any particular websites.

**Data Analysis**

Data from completed questionnaires were entered into a Microsoft Access database and SPSS data analysis software (Version 9.0) was used to apply statistical tests to the data. A p value of < 0.05 was used to indicate statistical significance. General Practitioner and Practice Nurse groups were compared using the chi-squared (chi²) test for nominal data, and the two sample t-test and Mann Whitney U tests for ordinal data. When differences between the two staff groups were demonstrated to be statistically significant, analysis of variance tests (ANOVA) were used to explore relationships.

## Results

A total of 160 of 300 completed questionnaires were returned by GPs, a response rate of 54%. There were 96 of 130 completed forms returned by Practice Nurses, providing a response rate of 74%. The overall response rate for the total population was 60% (N=256).

No evidence of a difference in age was found between the two staff groups. While the GPs were reasonably evenly distributed between the sexes (Male 55%:Female 45%), Practice Nurses were overwhelmingly female (Male 1%:Female 99%). The Internet is accessed by 79% of GPs and 73% of Practice Nurses. Primary Care Staff were asked from where they accessed the Internet (Table 1). Results show that significantly more GPs access the Internet from home (p=0.003). Overall, significantly more male Primary Care Staff access the Internet (p=0.001).

**Table 1:** Where do Primary Care Staff Access the Internet?

	**Practice** (No. of Staff)	**Home** (No. of Staff)
General Practitioner	108 (67.5%)	90 (56.3%)
Practice Nurse	60 (62.5%)	34 (35.4%)

**Table 2:** What Information Have You Found Useful as a Primary Care Clinician?

	**General Practitioner** (No. of Staff)	**Practice Nurse** (No. of Staff)
Disease Information	79 (49.7%)	44 (48.2%)
Online Journals	67 (42.1%)	34 (35.4%)
New Medical Information	47 (29.6%)	27 (29.0%)
Drug Information	37 (23.3%)	20 (20.8%)
Research Information	19 (11.9%)	19 (19.8%)
Other Information	8 (5.0%)	19 (10.6%)
Email with Other Clinicians	26 (16.4%)	4 (4.2%)
Complimentary Medicine	9 (5.7%)	3 (3.1%)
Healthcare Newsgroups	6 (3.8%)	3 (3.1%)
Notice of Meetings	10 (6.3%)	3 (3.1%)

Time (20%) and lack of skills (17%) were the most common reasons cited for not accessing the Internet (Table 2). Of those staff with Internet access, 69% of GPs and 70% of Practice Nurses had used it to access healthcare information. Table 2 shows which categories of information they found useful as Primary Care Clinicians. Significantly more GPs used the Internet for email with other clinicians (p=0.004). Primary Care Staff aged under 40 are more likely to refer to the Internet for drug information (p=0.03).

Staff were asked their opinion about the quality/accuracy of Internet healthcare information; 48% (74) of GPs and 41% (37) of Practice Nurses expressed concern over the reliability of information available via the Internet (Table 3).

Results show that 58% (91) of GPs and 34% (32) of Nurses have, at some time, been approached by patients with information about their condition obtained from the Internet. This showed a significant statistical difference between the 2 staff groups (p<0.001). On average, GPs have seen 2.9 patients holding Internet healthcare information in the past six months (min.=1, max.=20) and Practice Nurses 1.9 patients in the same period (min.=1, max.=6).

Table 3 outlines how Primary Care Staff interact with these patients. Surprisingly, 65% of the information presented by these patients was new to GPs, and only 45% of the patients had correctly interpreted the information in the GPs opinion. Both Staff Groups reported that the consultation time was increased, and significantly more Practice Nurses felt that they were able to use the consultation time more effectively (p=0.03).

**Table 3:** Consultation with Patients Holding Internet Healthcare Information

	**General Practitioner** (No. of Staff)	**Practice Nurse** (No. of Staff)
The patient participates more actively in his treatment	65 (78.3%)	26 (83.9%)
The patient has higher expectations	75 (85.2%)	26 (78.8%)
The information is accurate	59 (73.8%)	24 (75%)
The length of consultation is increased	68 (77.3%)	24 (72.7%)
This type of patient is a welcome challenge	46 (55.4%)	24 (72.7%)
The consultation is more interactive than usual	43 (50.6%)	22 (68.8%)
The patient correctly interpreted information	38 (44.7%)	19 (59.4%)
The patient is more demanding	50 (58.8%)	14 (42.4%)
The information is new to the clinician	55 (64.7%)	13 (40.6%)
The clinician was able to use the time more effectively	16 (19.0%)	12 (38.7%)

Primary Care Staff were asked to rate how they felt about patients obtaining information from the Internet (Table 4). Results highlight that significantly more GPs were indifferent to patients retrieving healthcare information from the Internet (chi²=5.42 DF=1 p=0.002), and significantly more Practice Nurses felt unsure about this issue (chi²=11.52 DF=1 p=0.001).

**Table 4:** How Do You Feel about Patients Obtaining Information from the Internet?

	**General Practitioner** (No. of Staff)	**Practice Nurse** (No. of Staff)
Positive	61 (39.1%)	29 (30.9%)
Indifferent	53 (34.0%)	19 (20.0%)
Uncomfortable	19 (12.2%)	15 (16.0%)
Not Sure	23 (14.7%)	31 (33.0%)

Almost half of the GPs, 45% (69), said they would consider referring a patient to the Internet for further information compared to only 29% (27) of the Practice Nurses. This was a statistically significant difference (p=0.01).

## Discussion

A recent study in Australia by Young and Ward [7] reported that 43% of GPs in New South Wales have access to the Internet at home or work. A survey of GPs in the United Kingdom in 1997 by Roscoe [3] showed that around 50% had Internet access. It is encouraging to note that in Glasgow, with recent government funding, this figure has now reached 79%. Lack of time is most commonly cited as a barrier to accessing the Internet; one GP commented: "Most days having lunch is a luxury, if a patient has the time let them use it". The other most commonly cited barrier was the lack of appropriate skills, which may point towards a need for education and continued professional development in this area. The following comment is typical of those Primary Care Staff who said they were of unsure of the technology: "Tend to get lost in it and it wastes a lot of time."

Approximately half of all the Primary Care Staff questioned have seen patients who supplemented their consultation with information obtained from the Internet. Results from this survey show that these patients have overwhelmingly higher expectations than average and more actively participate in their treatment (Table 3). With the continued proliferation of health sites on the Internet and more patients empowered by new personal computers and free Internet access, this type of patient consultation will increase [8]. The role of the clinician as patient advisor/interpreter of medical Internet information is a new and time-consuming task. This study does not address the actual time invested by clinicians in Internet related activities; but as Table 3 shows, 74% of clinicians identified that the length of consultation with patients holding Internet healthcare information was increased.

A large amount of the information collated from the Internet and presented by patients at their consultation is new to the clinician and, in some cases, it seems that practitioners do not view this as a welcome challenge. Results from this survey show that patients are having problems correctly interpreting the medical information they have downloaded. Comments from participants highlight the difficulty in assessing the quality of this information, its source, evidence-base, and date posted. One General Practitioner likened Internet healthcare sites to the Curate's egg - "good in parts." This would seem to reinforce the idea that healthcare sites should be given a "seal of approval," confirming they have been reviewed and deemed suitable for the general public. Indeed, many frameworks now exist to allow professionals to assess the credibility, accuracy, and relevance of healthcare information on the Internet [9], but clear, understandable rating criteria to assist the public in evaluating health related Internet sites are difficult to find.

As one may expect, those Primary Care Staff that have themselves accessed the Internet for healthcare information are significantly more likely to refer patients to the Internet for similar information. Primary Care Staff presented with new and accurate Internet healthcare information are also statistically more likely to refer a patient to the Internet.

In summary, if Primary Care Staff have a positive approach to the Internet themselves and have had good interactions with this type of patient, they appear far more open to the concept of this new technology and happier to refer their patients for more healthcare information.

This information will provide a basis for determining the current scope of these activities in Glasgow, and assist in determining future directions for preparing staff to deal more effectively with more knowledgeable patients.
